# The relative incidence of COVID-19 in healthcare workers versus non-healthcare workers: evidence from a web-based survey of Facebook users in the United States

**DOI:** 10.12688/gatesopenres.13202.2

**Published:** 2021-07-20

**Authors:** Abraham D. Flaxman, Daniel J. Henning, Herbert C. Duber

**Affiliations:** 1 Institute for Health Metrics and Evaluation, University of Washigton, Seattle, WA, 98195, USA; 2 Department of Emergency Medicine, University of Washigton, Seattle, WA, 98195, USA

**Keywords:** COVID-19, healthcare workers

## Abstract

**Background:** Healthcare workers are at the forefront of the COVID-19 pandemic and it is essential to monitor the relative incidence rate of this group, as compared to workers in other occupations. This study aimed to produce estimates of the relative incidence ratio between healthcare workers and workers in non-healthcare occupations.

**Methods:** Analysis of cross-sectional data from a daily, web-based survey of 1,822,662 Facebook users from September 8, 2020 to October 20, 2020. Participants were Facebook users in the United States aged 18 and above who were tested for COVID-19 because of an employer or school requirement in the past 14 days. The exposure variable was a self-reported history of working in healthcare in the past four weeks and the main outcome was a self-reported positive test for COVID-19.

**Results:** On October 20, 2020, in the United States, there was a relative COVID-19 incidence ratio of 0.73 (95% UI 0.68 to 0.80) between healthcare workers and workers in non-healthcare occupations.

**Conclusions:** In fall of 2020, in the United States, healthcare workers likely had a lower COVID-19 incidence rate than workers in non-healthcare occupations.

## Introduction

In August, the Peterson-KFF Health System Tracker published a collection of charts showing how healthcare utilization has declined during the COVID-19 pandemic in the United States ^1^, showing that facility discharge volume dropped by over 25% and cancer screening volumes dropped by over 85% from levels in 2019. This decrease is consistent with evidence from other sources ^2, 3^, and could be driven by a perceived risk of interacting with workers at health facilities. It is yet to be seen how much this delayed and foregone care will reduce population health. Meanwhile, a Wall Street Journal analysis of Centers for Disease Control and Prevention (CDC) data found that at least 7,400 COVID-19 infections were transmitted in US hospitals in 2020 ^4^. Access to adequate resources for infection prevention among health care workers (HCWs) remains a topic of urgent importance ^5^.

The existing evidence quantifying the relative COVID-19 incidence rate among HCWs as compared to workers in non-healthcare occupations (non-HCWs) has focused on the first wave of the pandemic, and found that HCWs are at higher risk of COVID ^6– 9^. We hypothesized that by fall of 2020 there was not a substantially elevated rate of COVID-19 infection among HCWs and that HCWs might even have lower incidence rate than non-HCWs, and we analyzed data from a large survey of Facebook users to investigate.

## Methods

### Study design

We analyzed individual participant data from a large, web-based survey of Facebook users aged 18 and above in the United States (around 300,000 respondents per week). Every day Facebook offered a random sample of US-based users a Qualtrics survey run by the Delphi lab at Carnegie Mellon University who made it rapidly available to other academic researchers ^10, 11^. Facebook also provided survey weights to adjust for non-response probability and to match the age and sex distribution at the national level ^12, 13^. This sort of survey data has been used previously to perform population based analyses related to COVID-19, though never before at such large scale ^14, 15^. Our analysis relied on the responses to two lines of questions: (1) questions about recent work history, worded as, “In the past 4 weeks, did you do any kind of work for pay?” and if so, “[p]lease select the occupational group that best fits the main kind of work you were doing in the last four weeks”; and (2) questions about COVID-19 testing history, worded as, “Have you **ever** been tested for coronavirus (COVID-19)?”, “[h]ave you been tested for coronavirus (COVID-19) in the **last 14 days**?”, “[d]id this test find that you had coronavirus (COVID-19)”, and “[d]o any of the following reasons describe why you were tested for coronavirus (COVID-19) in **the last 14 days**? Please select all that apply.”

We analyzed the six weeks of data from September 8, 2020 to October 20, 2020, which provided more than 80% power to detect a 30% difference between COVID-19 incidence in HCWs and non-HCWs.

### Variables

 To quantify the relative risk of COVID-19 among healthcare workers (HCWs) versus workers in non-healthcare occupations (non-HCWs), we used the response to the occupational group question as our exposure variable (we coded respondents who selected option “Healthcare practitioners and technicians” or “Healthcare support” as HCWs, and all others, including those with a missing value, as non-HCWs). We identified individuals with COVID-19 as those who reported that they had tested positive for COVID-19 in the last 14 days.

### Statistical methods

We calculated the endorsement rate of positive COVID-19 test (ER) for the HCW and non-HCW population as the survey-weighted percent of respondents in either group who reported COVID-19, and calculated the relative COVID-19 incidence ratio (RR) with the equation

   RR = (ER among HCWs) / (ER among non-HCWs).

We quantified the uncertainty in this ratio using non-parametric bootstrap resampling to obtain a 95% uncertainty interval ^16^. To control for confounding due to differential access to COVID-19 testing, we restricted our analysis to only HCWs and non-HCWs who were tested in the last 14 days because their employer or school required it.

As sensitivity analyses, we considered also alternative inclusion criteria and more restrictive subsets of HCWs. The survey provided survey weights that adjust for non-response bias, which we used in our main analysis. However, these weights were designed to represent the national population, and therefore might not represent the HCW population as accurately. As a sensitivity analysis, we repeated our calculation using the unweighted data. To investigate the possibility that workplace testing practices differ between HCW and non-HCW occupational settings, we also repeated our analysis with additional filtering based on the “why you were tested” question. In the main result we used the subset of individuals who responded that they were tested in the last 14 days because of employer/educational requirements, and this question has a “select all that apply” answer type, and also includes “I felt sick” as an option. As a sensitivity analysis, we used only those individuals who were tested because of a workplace requirement and did not feel sick.

### Ethical statement

These research activities used no identifiable private information and were therefore exempt from institutional board review.

## Results

The survey data contained 43,430 respondents who were tested due to workplace requirements in the time period we focused on, 14,660 HCWs and 28,770 non-HCWs (see Table 1 for demographic details). There were 2,145 respondents who reported a positive test for COVID-19 in the last 14 days (588 among HCWs and 1,557 among non-HCWs).

**Table 1.** Characteristics of survey respondents.

	Non- healthcare workers		Healthcare workers	
	n	(%)	n	(%)
**Total**	1,699,214	100.0	123,448	100.0
**Tested in last 14 days**	133,533	7.9	22,594	18.3
**Test required by work or school**	28,770	1.7	14,660	11.9
**Among those with required test**				
**Male gender**	9,303	32.3	2,106	14.4
**Age in years**				
**18 to 24**	3,595	12.5	818	5.6
**25 to 34**	4,994	17.3	2,544	17.4
**35 to 44**	5,146	17.9	3,255	22.2
**45 to 54**	5,179	18.0	3,587	24.5
**55 to 64**	4,227	14.7	3,345	22.8
**65 to 74**	1,307	4.5	976	6.7
**75 and older**	503	1.7	121	0.8

Among HCWs with a required test, 588 of 14,660 (4.0%) reported a positive test in the last 14 days, while among non-HCWs with a required test, 1,557 of 28,770 (5.4%) reported a positive test, for a relative COVID-19 incidence ratio of 0.73 (95% UI 0.68 to 0.80) ( Table 2).

**Table 2.** Relative COVID-19 incidence rate (RR) and counts of healthcare workers and non-healthcare workers and their crude counts and rates.

Healthcare workers			Non-healthcare workers				
Tested	Positive	%	Tested	Positive	%	RR	95% UI
14,660	588	4.0	28,770	1,557	5.4	0.73	0.68 to 0.80

Our power calculation simulation results showed that 7,000 simulants provide 80% power to reject a null hypothesis that HCWs and non-HCWs have the same RR if, in truth, the RR is 0.7. Since the survey currently collects a weekly volume of around 7,000 individuals who report taking a required COVID-19 test, the simulation results imply that six weeks of data will provide more than sufficient power.

### Sensitivity analyses

When we repeated our calculation using the unweighted survey responses to calculate the COVID-19 incidence ratio, we found nearly identical relative incidence ratio of 0.74 (95% UI 0.69 to 0.79).

When we repeated our analysis restricted to only specific subtypes of HCWs, as afforded by the questionnaire, we found a range of risks, usually less than 1.0, with substantially less certainty due to small sample sizes ( Table 3).

**Table 3.** Relative COVID-19 incidence rate (RR) and counts of healthcare workers (HCWs) and non-healthcare workers stratified by worker subtype.

HCW subtype	Number of non- subtype HCWs	Number of subtype HCWs	Relative risk	Lower bound	Upper bound
All HCWs	28,770	14,660	0.73	0.69	0.80
Physician or surgeon	43,139	291	2.71	1.86	3.60
Registered nurse (including nurse practitioner)	40,262	3,168	0.66	0.62	0.82
Licensed practical or licensed vocational nurse	41,318	2,112	0.73	0.60	0.86
Physician assistant	43,274	156	0.63	0.33	1.13
Dentist	43,392	38	0.85	0.24	2.22
Any other treating practitioner	43,046	384	0.56	0.31	0.81
Pharmacist	43,345	85	0.28	0.08	0.72
Any therapist	42,165	1,265	0.51	0.37	0.63
Any health technologist or technician	41,841	1,589	1.01	0.79	1.17
Veterinarian	43,395	35	0.29	0.00	1.28
Nursing assistant or psychiatric aide	41,812	1,618	1.02	0.80	1.22
Home health or personal care aide	42,847	583	0.77	0.52	1.00
Occupational or physical therapy assistant or aide	43,350	80	1.47	0.80	2.31
Massage therapist	43,426	4	10.16	0.00	13.21
Dental assistant	43,412	18	0.00	0.00	0.00
Medical assistant	43,280	150	1.25	0.64	1.96
Medical transcriptionist	43,402	28	0.56	0.00	1.38
Pharmacy aide	43,413	17	0.00	0.00	0.00
Phlebotomist	43,397	33	2.75	0.63	4.06
Veterinary assistant	43,422	8	1.74	0.00	6.97
Any other healthcare support worker	41,104	2,326	0.55	0.46	0.66

When we used only those individuals who were tested because of a workplace requirement *and* did not feel sick, we obtained a relative risk closer to 1.0. Using only those tested because of a workplace requirement who also *did* feel sick we still obtained a relative risk substantially smaller than 1.0 ( Table 4). Although this finding could suggest that differences in testing patterns between healthcare and other work settings are partially responsible for the different positivity rates among HCWs and non-HCWs, it could also be driven by greater access to COVID-19 testing for confirmation of illness among HCWs experiencing symptoms. The recall period of 14 days provides ample time for an individual to receive a workplace test without symptoms, then develop symptoms, and then receive another test to determine if the symptoms are due to COVID-19, and HCWs might have more opportunity to access such a follow-up test, since they are visiting a healthcare setting for work already.

**Table 4.** Relative COVID-19 incidence rate (RR) and counts of healthcare workers and non-healthcare workers stratified by those who reported they felt/did not feel sick as an additional reason for getting tested.

	Number of non-HCWs	Number of HCWs	Relative risk	Lower bound	Upper bound
Test required, did not feel sick	25,236	13,610	1.09	1.01	1.27
Test required, felt sick	3,534	1,050	0.80	0.69	0.92

## Discussion

This study utilized a population-based approach to examine the relative risk of COVID-19 infection among HCW compared with non-HCW. We founda relative COVID-19 incidence ratio substantially and significantly less than 1.0, which can be cautiously interpreted as a positive result, indicating that infection control measures being taken by HCWs in Fall of 2020 were effective.

Our findings are consistent with the limited other evidence available on the risk of COVID-19 in healthcare facility settings ^17– 20^, although also contrast with evidence from prior research that has found that HCWs are at higher risk of COVID ^6– 9^. This outbreak and our understanding of it have both changed rapidly in the past, and may do so again, so we will continue to update this information.

### Limitations

This work has at least three limitations. First, our results are based on self-reported data from a sample of Facebook users and therefore subject to both recall bias and social desirability bias, and may not be representative of the general population or the HCW population. The questions we relied on did not seem particularly at risk for these biases, although the question “have you been tested for COVID-19 in the last 14 days?” likely included positive responses from individuals who received seroprevalence testing as well as PCR testing, which could also introduce a small amount of bias; using this 14-day recall period as a proxy for incidence of COVID-19 could also introduce a small amount of bias. The impact of nonresponse bias is harder to gauge, however; our sensitivity analysis shows that the survey weights do influence our results. Second, our approach required a large sample size to obtain a sufficiently precise estimate of RR, but this seems safer than including respondents who did not report receiving a required test, as that could introduce confounding. Third, it is possible that there was still uncontrolled confounding due to differential access to tests between HCWs and non-HCWs. Our sensitivity analysis found substantively similar results when restricted only to individuals who had workplace testing when they did not feel sick, but since we have only considered respondents with tests required by their employer or school, this might focus on non-HCW setting with better-than-average infection control policies (for example, they *are* doing asymptomatic testing) and therefore the relative risk for HCWs might be even lower than our method estimated.

## Conclusion

In October, 2020, in the United States the relative infection ratio of HCWs to non-HCWs was lower than 1.0. Infection control remains essential and HCWs must continue to be protected as the COVID-19 pandemic continues, to ensure safety to themselves, their co-workers, and their patients.

## Data availability

### Underlying data

The underlying data used in this study are available to academic researchers for research purposes from Facebook at: https://www.facebook.com/research-operations/rfp/?title=covid19-symptom-survey-data-access. Conditions of access and instructions for applications can be found at https://dataforgood.fb.com/docs/covid-19-symptom-survey-request-for-data-access/.

## Code availability

Reproducibility code available from: https://github.com/aflaxman/covid_hcw_rr

Archived code at time of resubmission: https://doi.org/10.5281/zenodo.4270367 ^21^.

License: GNU General Public License v3.0
