# Supplementary material for: The relative incidence of COVID-19 in healthcare workers versus non-healthcare workers: evidence from a web-based survey of Facebook users in the United States
Source: Gates Open Res. 2021 Jul 20;4:174. Originally published 2020 Nov 27. [Version 2] doi: 10.12688/gatesopenres.13202.2 (PMC8355954; doi:10.12688/gatesopenres.13202.2)
Supplement: Peer review history

# Peer review: Reviewer response for version 2

**DOI:** 10.21956/gatesopenres.14528.r30926

**Published:** 2021-08-10

**Authors:** Devan Hawkins, Marcy Goldstein-Gelb

**Affiliations:** 1 Department of Public Health Program, Schools of Arts and Sciences, MCPHS University, Boston, MA, USA; 2 National Council for Occupational Safety and Health, Somerville, MA, USA

Thank you for this invitation to re-review the manuscript. We believe that the authors have done an excellent job addressing most of the major concerns. However, we are still concerned about how representative the population from the Facebook survey is of the population of healthcare workers. While there is publicly available data that could be used for comparisons, if the authors do not wish to include this comparison, we recommend that the authors add a note about this limitation.

Is the work clearly and accurately presented and does it cite the current literature?

Partly

If applicable, is the statistical analysis and its interpretation appropriate?

Partly

Are all the source data underlying the results available to ensure full reproducibility?

Yes

Is the study design appropriate and is the work technically sound?

Partly

Are the conclusions drawn adequately supported by the results?

Partly

Are sufficient details of methods and analysis provided to allow replication by others?

Yes

Reviewer Expertise:

NA

We confirm that we have read this submission and believe that we have an appropriate level of expertise to confirm that it is of an acceptable scientific standard.

# Peer review: Reviewer response for version 2

**DOI:** 10.21956/gatesopenres.14528.r30927

**Published:** 2021-07-29

**Authors:** Alex Reinhart

**Affiliations:** 1 Department of Statistics & Data Science, Carnegie Mellon University, Pittsburgh, PA, USA

I thank the authors for their careful revisions. These revisions address all of my comments in the initial round of review. I still found Table 3 slightly confusing (I initially interpreted "Number of non- subtype HCWs" to mean HCWs not of the subtype, rather than including all respondents, including non-HCWs), but otherwise I believe the revisions have improved the manuscript.

Is the work clearly and accurately presented and does it cite the current literature?

Yes

If applicable, is the statistical analysis and its interpretation appropriate?

Yes

Are all the source data underlying the results available to ensure full reproducibility?

Yes

Is the study design appropriate and is the work technically sound?

Yes

Are the conclusions drawn adequately supported by the results?

Yes

Are sufficient details of methods and analysis provided to allow replication by others?

Yes

Reviewer Expertise:

NA

I confirm that I have read this submission and believe that I have an appropriate level of expertise to confirm that it is of an acceptable scientific standard.

# Peer review: Reviewer response for version 1

**DOI:** 10.21956/gatesopenres.14411.r30426

**Published:** 2021-04-01

**Authors:** Tim Driscoll

**Affiliations:** 1 School of Public Health, Faculty of Medicine and Health, The University of Sydney, Sydney, NSW, Australia

This paper presents an analysis of data collected from United States’ respondents to a Facebook survey and focuses on a comparison of the rate of COVID-19 in health care workers compared to workers in other sectors. The main finding was that infection is less common in health care workers compared to non-health care workers, with the authors concluding that the results suggest it is “safe” (in terms of risk of COVID-19 infection) to be a health care worker. The methodology seems appropriate. The structure of the paper is good and the meaning is generally clear.

 In terms of the Methods, there are inconsistencies in the terminology and I can’t see any reason for this. Most particularly, there is mention of an “ *endorsement rate*”, which is the basis of the “ *relative COVID-19 incidence ratio*”, but this endorsement rate is not mentioned again in the manuscript. In the Results section, there is mention of a “ *relative COVID-19 prevalence ratio*” and a “ *Relative COVID-19 incidence rate*”. In the Discussion, “ *relative COVID-19 incidence ratio*” is mentioned again. I presume all three of these terms represent the same quantity. If so, it seems just one term should be used. If not, there needs to be further explanation about what has been calculated and why. It appears that the information presented is prevalence rather than incidence, because although the testing was in the previous 14 days the positive result could reflect past disease, depending on the type of test. If it is assumed the testing was done via PCR and further assumed this PCR test would only be positive for recent (in the previous two weeks or so) infection, then incidence would be an appropriate term to use, but then the implications of this assumption should be considered in the Discussion. Either way, the uncertainty arising from lack of information about the testing seems to be a limitation that could usefully be included at the end of the Discussion.

 The conclusion that “ *HCWs need not fear contracting or transmitting infections more than other workers do…*” seems too strong given the limitations of the data used for this study and the “ *…limited other evidence available…*”, as acknowledged by the authors. Similarly, the preceding statement that the result is “ *…an unequivocally positive finding…*” is at odds with the limitations considered later in the paper. I agree that if the results are accepted on face value they imply that health care workers are at lower risk than non-health care workers, but the other aspects just mentioned mean that conclusions based on these results should be guarded. Also, health care workers are analysed as a group, or in smaller but still broad groups in Table 3. This group will contain a mixture of people working directly with the public (front-line health workers) in a clinical setting and people working in health care but with minimal contact with patients. It might well be that the front-line health workers do indeed have a higher risk of infection than the general public, but that this is not reflected in the study results because the other health care workers have a much lower risk of infection. The fact that the “ *Physician or surgeon*” group appears to have a higher risk (RR=2.6) supports this concern. Having mentioned Table 3, the interpretation of this is not clear. Why are there different numbers of non-health care workers in each row, and why do they appear in any row if each row represents a different type of health care worker? It would be helpful to explain this.

 There is quite a bit of space in the paper considering the power of the study. The reason for this is not clear. The power calculations are based on an assumed difference of at least 30% in the “prevalence” of COVID-19 between health care workers and non-health care workers. This would be important if the difference found was less than 30%. However, since the difference found was 30%, the power calculations don’t seem relevant.  Also, the program to undertake this power calculation was included in the paper. I am not sure this adds much; I don’t mind it being there but it is not further considered and in fact isn’t directly referred to – it just appears in the text at the end of, or actually part of, the last sentence in the section describing the power calculation. That seems odd.

 The authors rightly identify some limitations in their work. These primarily result from the data used in the analysis rather than from the analysis used. The authors note the potential for some forms of reporting bias and for uncontrolled confounding, both of which I agree may be of concern.  They also mention the need for a large sample size, which doesn’t seem to be a limitation in terms of interpreting the results of the study; the large sample size is not a source of bias, just something that requires greater statistical resources.

Is the work clearly and accurately presented and does it cite the current literature?

Yes

If applicable, is the statistical analysis and its interpretation appropriate?

Yes

Are all the source data underlying the results available to ensure full reproducibility?

Yes

Is the study design appropriate and is the work technically sound?

Yes

Are the conclusions drawn adequately supported by the results?

Partly

Are sufficient details of methods and analysis provided to allow replication by others?

Yes

Reviewer Expertise:

Epidemiology, occupational medicine

I confirm that I have read this submission and believe that I have an appropriate level of expertise to confirm that it is of an acceptable scientific standard, however I have significant reservations, as outlined above.

# Response

**Published:** 2021-05-17

**Authors:** Abraham Flaxman

**Affiliations:** University of Washington, Seattle, USA

*In terms of the Methods, there are inconsistencies in the terminology and I can’t see any reason for this. Most particularly, there is mention of an “endorsement rate”, which is the basis of the “relative COVID-19 incidence ratio”, but this endorsement rate is not mentioned again in the manuscript. In the Results section, there is mention of a “relative COVID-19 prevalence ratio” and a “Relative COVID-19 incidence rate”. In the Discussion, “relative COVID-19 incidence ratio” is mentioned again. I presume all three of these terms represent the same quantity. If so, it seems just one term should be used. If not, there needs to be further explanation about what has been calculated and why. It appears that the information presented is prevalence rather than incidence, because although the testing was in the previous 14 days the positive result could reflect past disease, depending on the type of test. If it is assumed the testing was done via PCR and further assumed this PCR test would only be positive for recent (in the previous two weeks or so) infection, then incidence would be an appropriate term to use, but then the implications of this assumption should be considered in the Discussion. Either way, the uncertainty arising from lack of information about the testing seems to be a limitation that could usefully be included at the end of the Discussion.*

**Response: **We have standardized our terminology on incidence, which we think is the most precise and accurate of the terms we used originally; thank you for calling attention to this inconsistency.  We have also added to the limitations section to highlight the way 14-day recall is not exactly “incidence”.

*The conclusion that “HCWs need not fear contracting or transmitting infections more than other workers do…” seems too strong given the limitations of the data used for this study and the “…limited other evidence available…”, as acknowledged by the authors. Similarly, the preceding statement that the result is “…an unequivocally positive finding…” is at odds with the limitations considered later in the paper. I agree that if the results are accepted on face value they imply that health care workers are at lower risk than non-health care workers, but the other aspects just mentioned mean that conclusions based on these results should be guarded. Also, health care workers are analysed as a group, or in smaller but still broad groups in Table 3. This group will contain a mixture of people working directly with the public (front-line health workers) in a clinical setting and people working in health care but with minimal contact with patients. It might well be that the front-line health workers do indeed have a higher risk of infection than the general public, but that this is not reflected in the study results because the other health care workers have a much lower risk of infection. The fact that the “Physician or surgeon” group appears to have a higher risk (RR=2.6) supports this concern.*

**Response: **We have moderated the discussion in light of this comment, as well as the similar concerns from Reviewer 2.

*Having mentioned Table 3, the interpretation of this is not clear. Why are there different numbers of non-health care workers in each row, and why do they appear in any row if each row represents a different type of health care worker? It would be helpful to explain this.*

**Response: **Each row besides the first row compares a subtype of HCWs to everyone who is not of that subtype.  We have edited the column headings to make this clearer.

*There is quite a bit of space in the paper considering the power of the study. The reason for this is not clear. The power calculations are based on an assumed difference of at least 30% in the “prevalence” of COVID-19 between health care workers and non-health care workers. This would be important if the difference found was less than 30%. However, since the difference found was 30%, the power calculations don’t seem relevant.  Also, the program to undertake this power calculation was included in the paper. I am not sure this adds much; I don’t mind it being there but it is not further considered and in fact isn’t directly referred to – it just appears in the text at the end of, or actually part of, the last sentence in the section describing the power calculation. That seems odd.*

**Response: **We did this power calculation in so much detail because we wanted to get our results out as soon as possible, but not so soon that we were fooled by chance variation in the data.  We have taken it out to focus the reader on the most important parts, especially now that there is so much more data available.

*The authors rightly identify some limitations in their work. These primarily result from the data used in the analysis rather than from the analysis used. The authors note the potential for some forms of reporting bias and for uncontrolled confounding, both of which I agree may be of concern.  They also mention the need for a large sample size, which doesn’t seem to be a limitation in terms of interpreting the results of the study; the large sample size is not a source of bias, just something that requires greater statistical resources.*

**Response: **We thank the reviewer for this perspective, and have attempted to edit the limitations section to make it clearer.

# Peer review: Reviewer response for version 1

**DOI:** 10.21956/gatesopenres.14411.r30475

**Published:** 2021-03-29

**Authors:** Devan Hawkins, Marcy Goldstein-Gelb

**Affiliations:** 1 Department of Public Health Program, Schools of Arts and Sciences, MCPHS University, Boston, MA, USA; 2 National Council for Occupational Safety and Health, Somerville, MA, USA

Thank you for the invitation to review this paper. The paper addresses an important topic (the risk of acquiring COVID-19 among healthcare workers). The authors apply unique methods to study the problem. However, we have some concerns about how the analysis was performed and how the results were interpreted. Below, we provide details about these concerns. 

 Introduction:

- The authors should provide some information about previous studies that have examined the risk for COVID-19 among healthcare workers and also justify why they hypothesized that healthcare workers would have a lower risk. Some studies have suggested that they have an elevated risk. Below are some studies that have examined the risk/potential risk for COVID-19 among healthcare workers:
  - Baker *et al.* (2020 ^1^).
  - Burrer *et al.* (2020 ^2^).
  - Hawkins *et al.* (2020 ^3^).
  - Ran *et al. *(2020 ^4^).

Methods:

- The authors should explain the justification for weighting to the overall Facebook population more. If the goal is to ensure that the healthcare workers survey from Facebook are representative of healthcare workers, this type of weighting may not help. 
- Was industry information available? There is good reason to suspect that risk will be different across different industry. In some cases, HCWs will even be working from home with telehealth. It may be useful to:
  - 1) Compare healthcare workers employed in the healthcare industry to other health care workers
  - 2) Examine the risk among different industries 
- We strongly recommend including all positive tests as a sensitivity analysis not just those required by work. I agree that differential testing may introduce a bias, but it would be better to show all the data so that we can consider the potential magnitude of that bias. There may actually be an even greater differential between HCW and other workers.  In fact, probably most non-health care workers don't get tested through employer requirements, and only know that they have COVID after becoming sick.
- Additionally, we strongly recommend having a different reference population than all non-healthcare workers. Other high risk workers are included in the current reference group, which may have the impact of making the risk among healthcare workers appear lower. Potentially consider including major census or SOC occupations for comparison. 
- For non-health care workers, did they ask whether they worked outside the home, or was there just an assumption that they did.  Naturally if they were tested but work from home, that would be an overrepresentation of work-relatedness, though I would assume it would not be an employer requirement if they work from home.
- Was the survey only conducted in English? 

Results:

- The demographics for healthcare workers should be compared to national data about healthcare workers demographics. This data can be obtained from the CPS or census. CPS is linked here: https://www.bls.gov/cps/tables.htm
- Consider separating occupations into major categories for more fair comparisons. You may consider weighting to this data rather than the Facebook demographics. 
- Is race/ethnicity data available? If workers of color are under-represented this could introduce bias to the study, because these workers may be more likely to be employed in higher risk healthcare occupations. 
- Table 3: How do the distributions of detailed occupations compare to national data about employment in these occupations? The CPS data linked above can be used to assess this. Bias may be introduced if certain occupations are underrepresented. 
- Table 3: The authors should discuss the variability in rates according to specific healthcare occupations. They may consider including the groups according to major healthcare occupations (practioners, support, etc.). Some occupations have elevated rates.  

Discussion:

- We strongly recommend removing this finding: “an unequivocally positive findings, indicating that infection control measures being taken by HCWs in total are effective.” Based on the limitations of this study, we do not believe that the findings support this conclusion. The findings may be suggestive of effective measures being taken if some of the limitations in the methods/results are addressed. 
- Consider other findings linked above which are not consistent with this study’s findings of a lower risk among HCWs.
- We strong discourage concluding that HCWs should not fear contracting or transmitting infections more than other workers. HCWs don't base their fear on how their likelihood of exposure compares to other worker fears - they're afraid, according to other factors, including often not having adequate protection methods. 

Is the work clearly and accurately presented and does it cite the current literature?

Partly

If applicable, is the statistical analysis and its interpretation appropriate?

Partly

Are all the source data underlying the results available to ensure full reproducibility?

Yes

Is the study design appropriate and is the work technically sound?

Partly

Are the conclusions drawn adequately supported by the results?

Partly

Are sufficient details of methods and analysis provided to allow replication by others?

Yes

Reviewer Expertise:

Devan Hawkins: Occupational health epidemiologist

We confirm that we have read this submission and believe that we have an appropriate level of expertise to state that we do not consider it to be of an acceptable scientific standard, for reasons outlined above.

## References

[1] : Estimating the burden of United States workers exposed to infection or disease: A key factor in containing risk of COVID-19 infection.*PLoS One*.2020;15(4) : 10.1371/journal.pone.0232452e023245210.1371/journal.pone.023245232343747PMC7188235

[2] : Characteristics of Health Care Personnel with COVID-19 — United States, February 12–April 9, 2020. *MMWR. Morbidity and Mortality Weekly Report*.2020;69(15) : 10.15585/mmwr.mm6915e6477-48110.15585/mmwr.mm6915e632298247PMC7755055

[3] : COVID-19 deaths by occupation, Massachusetts, March 1-July 31, 2020.*Am J Ind Med*.2021;64(4) : 10.1002/ajim.23227238-24410.1002/ajim.2322733522627PMC8013546

[4] : Risk Factors of Healthcare Workers With Coronavirus Disease 2019: A Retrospective Cohort Study in a Designated Hospital of Wuhan in China. *Clinical Infectious Diseases*.2020;71(16) : 10.1093/cid/ciaa2872218-222110.1093/cid/ciaa287PMC718448232179890

# Response

**Published:** 2021-05-17

**Authors:** Abraham Flaxman

**Affiliations:** University of Washington, Seattle, USA

*Introduction:*

- *The authors shouldprovide some information about previous studies that have examined the risk for COVID-19 among healthcare workers and also justify why they hypothesized that healthcare workers would have a lower risk. Some studies have suggested that they have an elevated risk. Below are some studies that have examined the risk/potential risk for COVID-19 among healthcare workers:*
- *1. Baker MG, Peckham TK, Seixas NS: Estimating the burden of United States workers exposed to infection or disease: A key factor in containing risk of COVID-19 infection.PLoS One. 2020; **15** (4): e0232452 **PubMed Abstract** | **Publisher Full Text*
- *2. CDC COVID-19 Response Team, CDC COVID-19 Response Team, Burrer S, de Perio M, et al.: Characteristics of Health Care Personnel with COVID-19 — United States, February 12–April 9, 2020. MMWR. Morbidity and Mortality Weekly Report. 2020; **69** (15): 477-481 **Publisher Full Text*
- *3. Hawkins D, Davis L, Kriebel D: COVID-19 deaths by occupation, Massachusetts, March 1-July 31, 2020.Am J Ind Med. 2021; **64** (4): 238-244 **PubMed Abstract** | **Publisher Full Text*
- *4. Ran L, Chen X, Wang Y, Wu W, et al.: Risk Factors of Healthcare Workers With Coronavirus Disease 2019: A Retrospective Cohort Study in a Designated Hospital of Wuhan in China. Clinical Infectious Diseases. 2020; **71** (16): 2218-2221 **Publisher Full Text*

**Response: **Thank you for calling our attention to this growing body of work. We have added to this introduction to include this prior work and clarify our hypothesis.

*Methods:*

- *The authors should explain the justification for weighting to the overall Facebook population more. If the goal is to ensure that the healthcare workers survey from Facebook are representative of healthcare workers, this type of weighting may not help. *

**Response: **Thank you for identifying this risk to the validity of our findings. We have added more detail about the weights in the Study Design section, as well as additional caveats about using the weights for the HCW population in sensitivity analyses in the Statistical Methods section. We have also added to the limitations section to provide more caveats about the risk of non-response bias.

*Was industry information available? There is good reason to suspect that risk will be different across different industry. In some cases, HCWs will even be working from home with telehealth. It may be useful to:*

- *1) Compare healthcare workers employed in the healthcare industry to other health care workers*
- *2) Examine the risk among different industries *

**Response: **Unfortunately, the survey instrument does not distinguish between occupation and industry, and therefore we can only examine risk between different occupations, as identified by responses to the question “[p]lease select the occupational group that best fits the main kind of work you were doing in the last four weeks”.  Respondents selected a single category from a short list, and then a detailed category from a longer list, and all of the detailed categories that of HCW are listed in Table 3.

*We strongly recommend including all positive tests as a sensitivity analysis not just those required by work. I agree that differential testing may introduce a bias, but it would be better to show all the data so that we can consider the potential magnitude of that bias. There may actually be an even greater differential between HCW and other workers.  In fact, probably most non-health care workers don't get tested through employer requirements, and only know that they have COVID after becoming sick.*

**Response: **The results of this proposed sensitivity analysis might surprise the reviewer: in an analysis of all survey respondents (123,448 HCWs and 1,699,214 non-HCWs) we find that among HCWs (tested and untested), 1,674 of 123,448 (1.4%) reported a positive test in the last 14 days; while among non-HCWs (tested and untested), 11,963 of 1,699,214 (0.70%) reported a positive test.  This yields a ratio of 1.8 (95% UI 1.52 to 2.03), but it is confounded by the fact that HCWs have greater access to testing than non-HCWs and cannot be used as an estimate of the relative incidence ratio of COVID-19.

 If we restrict our analysis to only individuals who have been tested in the last 14 days, we find 156,127 respondents who were tested (regardless of workplace requirements) in the time period we focused on, 22,594 HCWs and 133,533 non-HCWs; Among HCWs tested (regardless of whether the test was required), 1,674 of 22,594 (7.4%) reported a positive test in the last 14 days, while among non-HCWs tested (regardless of whether the test was required), 11,963 of 133,533 (8.96%) reported a positive test, for an RR of 0.8 (95% UI 0.78 to 0.83).

**Response: **We prefer to keep this complexity out of the main paper; in some occupations, required testing happens only after symptoms develop, and in light of this, we prefer our sensitivity analysis using only required tests among asymptomatic workers to investigating this potential risk of confounding.

*Additionally, we strongly recommend having a different reference population than all non-healthcare workers. Other high risk workers are included in the current reference group, which may have the impact of making the risk among healthcare workers appear lower. Potentially consider including major census or SOC occupations for comparison.*

**Response: **We prefer to focus our discussion on a comparison of HCWs with all non-HCWs, but the reviewer raises an interesting additional question.  Although we choose to leave a full investigation of these occupational comparisons for future work, we cannot resist examining them briefly in this response. After HCWs, the occupation with the highest rates of required testing are (16) Other occupation, (2) education, training, and library, (11) office and administration services, and (7) food preparation and serving. Our comparison of HCWs to workers in occupation "Other" found a relative COVID-19 incidence ratio of 0.97 (95% UI 0.82 to 1.12).

 This also identifies an important divergence between the “non-HCW” population and the worker population---there are 9,652 respondents without an occupation code included in the non-HCW population.  Repeating our analysis with these respondents excluded finds a ratio of 0.60 (95% UI 0.55 to 0.67).

*For non-health care workers, did they ask whether they worked outside the home, or was there just an assumption that they did.  Naturally if they were tested but work from home, that would be an overrepresentation of work-relatedness, though I would assume it would not be an employer requirement if they work from home.*

**Response: **The survey does include the question “Was any of your work for pay in the last four weeks outside your home?”, and as an additional sensitivity analysis which we excluded from our report we considered the same analysis stratified on work-from-home status. We were surprised to find quantitatively similar results among those who work from home and those who do not.

*Was the survey only conducted in English?*

 The survey was translated into multiple languages (Spanish, French, Portuguese, Chinese, Vietnamese).  We have added a reference to the https://cmu-delphi.github.io/delphi-epidata/symptom-survey/ website with full details on the survey instrument.

*Results:*

  

- *The demographics for healthcare workers should be compared to national data about healthcare workers demographics. This data can be obtained from the CPS or census. CPS is linked here: https://www.bls.gov/cps/tables.htm*

**Response: **We appreciate this suggestion, but prefer to keep the main paper simpler and instead include the comparison in this response only.  Among survey respondents, HCWs were 85.7% female, while among employed persons in 2020, “Healthcare practitioners and technical occupations” were 74.4% female.  The age distribution was also similar, but not identical.

*Consider separating occupations into major categories for more fair comparisons. You may consider weighting to this data rather than the Facebook demographics.*

**Response: **We agree that this would be a valuable extension of the approach we have applied in this paper, but we would like to limit the scope of this work to focus solely on the comparison of HCWs to non-HCWs, and leave further investigation and comparison of other occupations and categories for future work.  We agree that additional sensitivity analyses would be warranted in this future work to determine if alternative weighting of the data yields substantively divergent results.  We believe, however, that our sensitivity analyses for the HCW versus non-HCW comparison establish that the substantive finding of an RR substantially below 1.0 for HCWs is robust.

*Is race/ethnicity data available? If workers of color are under-represented this could introduce bias to the study, because these workers may be more likely to be employed in higher risk healthcare occupations.*

**Response: **The survey instrument did include race and ethnicity information, but we do not currently have access to these columns of the data. Subsequent work investigating racial and ethnic differences in both response rates and test results would be very interesting.

*Table 3: How do the distributions of detailed occupations compare to national data about employment in these occupations? The CPS data linked above can be used to assess this. Bias may be introduced if certain occupations are underrepresented.*

**Response: **Some of the age distributions are quite similar, for example for nurses, while others have small sample sizes and are probably biased by differential response patterns, for example physicians.  Though we included all subcategories for completeness, we felt it was important to include the sample size as well, to make sure readers were not overly influenced by the calculations based on only a small number of respondents.

 We agree that this would be a valuable extension of the approach we have applied in this paper, but we would like to limit the scope of this work to focus solely on the comparison of HCWs to non-HCWs, and leave further investigation and comparison of other occupations and categories for future work.

*Discussion:*

- *We strongly recommend removing this finding: “an unequivocally positive findings, indicating that infection control measures being taken by HCWs in total are effective.” Based on the limitations of this study, we do not believe that the findings support this conclusion. The findings may be suggestive of effective measures being taken if some of the limitations in the methods/results are addressed. *

**Response: **We appreciate the reviewers recommendation and we have substantially moderated the discussion to ensure we keep readers aware of the limitations of our approach and do not over-state the implications our findings.

*Consider other findings linked above which are not consistent with this study’s findings of a lower risk among HCWs.*

**Response: **We have referred to this contrasting evidence base in the discussion now, as well as in the introduction.

- We strong discourage concluding that HCWs should not fear contracting or transmitting infections more than other workers. HCWs don't base their fear on how their likelihood of exposure compares to other worker fears - they're afraid, according to other factors, including often not having adequate protection methods. 

**Response: **We have moderated the language in our conclusion, and thank the reviewer again for helping us avoid over-stating the implications of our findings.

# Peer review: Reviewer response for version 1

**DOI:** 10.21956/gatesopenres.14411.r30079

**Published:** 2020-12-04

**Authors:** Alex Reinhart

**Affiliations:** 1 Department of Statistics & Data Science, Carnegie Mellon University, Pittsburgh, PA, USA

This presents a timely and useful analysis of large-scale survey data. For an analysis like this, it's very important to clearly present the meaning of the data and the caveats in the survey design; the authors do a good job here, and my comments here focus on making the paper even clearer.

 The analysis seems reasonable overall, and, subject to the limitations of the survey design, a useful contribution to the area.

 I've separated my comments into "Main comments", which I think should be addressed to make the article more sound, and "Minor comments" that just make minor improvements to the paper.

**Main comments:**

- The "Sensitivity analyses" section (page 5) explains that "When we repeated our calculation using the unweighted survey responses to calculate the COVID-19 incidence ratio, we found an even smaller relative incidence ratio of 0.4 (95% UI 0.3 to 0.5)." This seems surprising. Do you have any hypotheses that could explain why this is? It suggests that either the age and gender distributions for HCWs and non-HCWs are quite different (since the survey weights correct for age and gender) or that the estimated non-response for the groups are quite different.
- The last paragraph of the Discussion suggests the possibility that "since we have only considered respondents with tests required by their employer or school, this might focus on non-HCW setting with better-than-average infection control policies". This may be a good subject for an additional table of results: A comparison of the distributions of occupation among non-HCW people who were required to be tested and those who were not. Such a table would tell the reader whether those who are required to be tested are from an unusual group of occupations, to help tell whether those occupations might be higher or lower risk than average.
- Table 3 contains a "Number of non-HCWs" column, but I don't know how to interpret this. What does it mean to say that there were 26,805 non-HCWs in the "All HCWs" row?
- In the Limitations (page 6), the authors mention recall bias and social desirability bias as possible problems. But another key bias would be response bias: while Facebook's weights try to adjust for non-response, if they do not completely adjust for every possible factor related to non-response, there can still be bias. For example, if people who are much more concerned about COVID and take more precautions are also more likely to participate in the survey, and if Facebook does not have covariates that can predict this accurately, the survey sample can be biased relative to the population. It would be good to address this and indicate how it could affect the results.

**Minor comments:**

- The "Study design" subsection mentions that "Facebook also provided survey weights to adjust for the demographics of the active Facebook user population." It would be good to be explicit about what corrections are included in the weights:
  - The weights adjust for non-response, using Facebook's estimate of the probability of each sampled individual participating in the survey.
  - The weights are then post-stratified by age and gender only.
- In the "Study design" subsection, the second paragraph states "We analyzed the most recently available six weeks of data from September 6, 2020 to October 18, 2020", but Wave 4 of the survey (containing the occupation and testing questions) was only deployed on September 8, 2020. If data from September 6 and 7 was included, I assume it was left out of the study, because the respondents would not have answered the relevant questions.
- It may help readers to be explicit about the survey text and its location. The survey documentation site contains the full text of each survey wave, and referring to this could help readers who want to read the survey text and flow.

Is the work clearly and accurately presented and does it cite the current literature?

Yes

If applicable, is the statistical analysis and its interpretation appropriate?

Yes

Are all the source data underlying the results available to ensure full reproducibility?

Yes

Is the study design appropriate and is the work technically sound?

Yes

Are the conclusions drawn adequately supported by the results?

Yes

Are sufficient details of methods and analysis provided to allow replication by others?

Yes

Reviewer Expertise:

I am a professional statistician and assistant teaching professor of Statistics & Data Science at Carnegie Mellon University. I am also a member of the Delphi group, and manage the collection of the survey data described in this article; see my Competing Interests for further details.

I confirm that I have read this submission and believe that I have an appropriate level of expertise to confirm that it is of an acceptable scientific standard, however I have significant reservations, as outlined above.

# Response

**Published:** 2021-05-17

**Authors:** Abraham Flaxman

**Affiliations:** University of Washington, Seattle, USA

*This presents a timely and useful analysis of large-scale survey data. For an analysis like this, it's very important to clearly present the meaning of the data and the caveats in the survey design; the authors do a good job here, and my comments here focus on making the paper even clearer.*

**Response: **We thank the reviewer for this assessment.

*The analysis seems reasonable overall, and, subject to the limitations of the survey design, a useful contribution to the area.*

*I've separated my comments into "Main comments", which I think should be addressed to make the article more sound, and "Minor comments" that just make minor improvements to the paper.*

***Main comments:***

- *The "Sensitivity analyses" section (page 5) explains that "When we repeated our calculation using the unweighted survey responses to calculate the COVID-19 incidence ratio, we found an even smaller relative incidence ratio of 0.4 (95% UI 0.3 to 0.5)." This seems surprising. Do you have any hypotheses that could explain why this is? It suggests that either the age and gender distributions for HCWs and non-HCWs are quite different (since the survey weights correct for age and gender) or that the estimated non-response for the groups are quite different.*

**Response: **This appears to be an error in our number-plugging!  In the archived code corresponding to this submission, we have a relative incidence ratio of 0.70 (95% UI 0.65 to 0.74). We apologize for this and thank the reviewer for their careful reading that helped find and fix this defect!

*The last paragraph of the Discussion suggests the possibility that "since we have only considered respondents with tests required by their employer or school, this might focus on non-HCW setting with better-than-average infection control policies". This may be a good subject for an additional table of results: A comparison of the distributions of occupation among non-HCW people who were required to be tested and those who were not. Such a table would tell the reader whether those who are required to be tested are from an unusual group of occupations, to help tell whether those occupations might be higher or lower risk than average.*

**Response: **We appreciate the reviewer’s suggestion, but prefer to restrict the scope of this paper to focus only on HCWs, and leave investigation of other occupations for future research.

*Table 3 contains a "Number of non-HCWs" column, but I don't know how to interpret this. What does it mean to say that there were 26,805 non-HCWs in the "All HCWs" row?*

**Response: **Thank you for flagging this confusing terminology.  By “non-HCWs” we meant the number of respondents who are *not *in the HCW subgroup for which the row reports the relative risk.  We have renamed the column headers to make this clearer.

- *In the Limitations (page 6), the authors mention recall bias and social desirability bias as possible problems. But another key bias would be response bias: while Facebook's weights try to adjust for non-response, if they do not completely adjust for every possible factor related to non-response, there can still be bias. For example, if people who are much more concerned about COVID and take more precautions are also more likely to participate in the survey, and if Facebook does not have covariates that can predict this accurately, the survey sample can be biased relative to the population. It would be good to address this and indicate how it could affect the results.*

**Response: **Thank you for calling attention to this important limitation.  We have added a sentence to the limitations section about it.

***Minor comments:***

- *The "Study design" subsection mentions that "Facebook also provided survey weights to adjust for the demographics of the active Facebook user population." It would be good to be explicit about what corrections are included in the weights:*
  -
    - *The weights adjust for non-response, using Facebook's estimate of the probability of each sampled individual participating in the survey.*
    - *The weights are then post-stratified by age and gender only.*

**Response: **We have edited to include this detail explicitly.

*In the "Study design" subsection, the second paragraph states "We analyzed the most recently available six weeks of data from September 6, 2020 to October 18, 2020", but Wave 4 of the survey (containing the occupation and testing questions) was only deployed on September 8, 2020. If data from September 6 and 7 was included, I assume it was left out of the study, because the respondents would not have answered the relevant questions.*

**Response: **Good point, we have updated to text to reflect the days use only Wave 4 data, and shifted the data end date to still include precisely 6 weeks of data. This resulted in minor changes to many of our results, but no changes to our substantive findings.

  

- *It may help readers to be explicit about the survey text and its location. The **survey documentation site** contains the full text of each survey wave, and referring to this could help readers who want to read the survey text and flow.*

**Response: **Thank you for suggesting this, we have added a reference to this documentation.
